# Report of two paediatric cases of central line infections caused by species of the genus *Kocuria*

**DOI:** 10.1099/jmmcr.0.005040

**Published:** 2016-06-25

**Authors:** Sejal Makvana Bhavsar, Camille L. Hamula, Tanis C. Dingle

**Affiliations:** ^1^​ Department of Pediatric Infectious Disease, Mount Sinai Hospital/Icahn School of Medicine at Mount Sinai, 1 Gustave L. Levy Place, New York, NY 10029, USA; ^2^​ Department of Pathology, Mount Sinai Hospital/Icahn School of Medicine at Mount Sinai, 1 Gustave L. Levy Place, New York, NY 10029, USA

**Keywords:** *Kocuria*, line infection, paediatiric, line removal

## Abstract

**Introduction:**

Species of the genus *Kocuria* are Gram-positive cocci of the family *Micrococcacceae* that are ubiquitous in the environment and part of the normal skin and oral flora in humans. A paucity of cases have been reported of *Kocuria* as human pathogens and there are currently no evidence-based guidelines for managing these uncommon infections.

**Case presentation:**

We present two paediatric cases of central line infections with species of the genus *Kocuria* that required line removal despite antimicrobial therapy.

**Conclusion:**

Species of the genus *Kocuria* are uncommon human pathogens that have rarely been reported to cause opportunistic infections in both adult and paediatric populations. The cases presented here add to the growing body of literature documenting the pathogenicity of these organisms and the possible need for line removal to achieve clinical cure in central line-associated bacteraemia caused by species of the genus *Kocuria*.

## Introduction

Species of the genus *Kocuria* are Gram-positive cocci of the family *Micrococcaceae* that are non-motile, catalase-positive and coagulase-negative. They are ubiquitous in the environment and part of the normal skin and oral flora of humans and other mammals (Purty *et al.*, 2013). The few cases that have been reported of members of the genus *Kocuria* as human pathogens describe infections usually affecting immunocompromised patients and patients with indwelling catheters. There are currently no evidence-based practice guidelines for managing these uncommon infections, nor guidelines for testing the antimicrobial susceptibility of these organisms *in vitro*. Here, we present two paediatric cases of *Kocuria* central line infections that occurred within a short time frame at our institution, both of which required line removal despite antimicrobial therapy.

## Case Reports

### Case 1

A 3-year-old female with a history of bilateral Wilms tumor status post nephrectomy and chemotherapy, and who was receiving haemodialysis and peritoneal dialysis while awaiting a living-related renal transplant, presented with a two day history of fever, emesis and decreased activity level. At the dialysis center, she was noted to have leukocytosis (39000 cells µl^−1^) and thus blood cultures were collected from her dialysis catheter and she was admitted to the inpatient floor and empirically started on vancomycin and gentamicin. Gentamicin was discontinued on hospital day 2 upon identification of a Gram-positive organism from a positive central line blood culture from admission. The organism was identified as *Kocuria kristinae* (VITEK 2; bioMérieux) and was persistently isolated from her dialysis catheter for six consecutive days despite treatment with vancomycin (MIC=1 mcg ml^−1^; Table 1). Her symptoms improved by hospital day 3 and she had her dialysis catheter removed on hospital day 7. Peripheral blood cultures sent thereafter were negative.

### Case 2

A 7-month-old male with a medical history significant for congenital intestinal atresia status post repair with subsequent short gut syndrome and requiring TPN, presented with fever for two days and no other symptoms. He had mild leukocytosis (16000 cells µl^−1^) on his complete blood count. Blood cultures were drawn from his broviac catheter and peripherally, and cefepime and vancomycin were started for presumed line infection. Cefepime was discontinued on hospital day 2 upon identification of a Gram-positive organism from blood cultures drawn both from his line and peripherally during his admission to the hospital. Thereafter, daily blood cultures were obtained. He defervesced on hospital day 2 but his blood cultures were continuously positive for *Kocuria varians* for six days despite treatment with vancomycin (MIC=0.5 mcg ml^−1^; Table 1). On hospital day 7, he had his broviac catheter removed and blood cultures sent thereafter from a new central line were negative.

**Table 1.** Minimum inhibitory concentrations (MIC; mcg ml^−1^) of the species of *Kocuria* isolated in Case 1 and Case 2 as determined by automated microbroth dilution (VITEK-2) or E-test Susceptibility testing was performed as per CLSI guidelines for *Staphylococcus* because interpretations are not available for species of the genus *Kocuria.*

Antibiotic	Case 1 *Kocuria kristinae*	Case 2 *Kocuria varians*
Ciprofloxacin	≤0.5 mcg ml^−1^	4 mcg ml^−1^
Clindamycin	0.5 mcg ml^−1^	0.25 mcg ml^−1^
Daptomycin	≤0.12 mcg ml^−1^	0.032 mcg ml^−1^
Erythromycin	≤0.25 mcg ml^−1^	2 mcg ml^−1^
Gentamicin	≤0.5 mcg ml^−1^	2 mcg ml^−1^
Levofloxacin	1 mcg ml^−1^	1 mcg ml^−1^
Linezolid	2 mcg ml^−1^	0.5 mcg ml^−1^
Oxacillin	1 mcg ml^−1^	0.064 mcg ml^−1^
Tetracycline	1 mcg ml^−1^	0.5 mcg ml^−1^
Trimethroprim/sulfamethoxazole	≤0.25 mcg ml^−1^	0.125 mcg ml^−1^
Vancomycin	1 mcg ml^−1^	0.5 mcg ml^−1^

## Discussion

There are presently 18 known species of the genus *Kocuria*, but only five have been recognized to cause opportunistic infections: *K. kristinae*, *K. varians*, *K. marina*, *K. rhizophila* and *K. rosea* (Savini *et al.*, 2010). Documented infections in humans are rare, possibly due to low pathogenicity or misidentification in the past as coagulase-negative *Staphylococcus* or *Micrococcus*, which are also catalase-positive, coagulase-negative, Gram-positive cocci in clusters (Savini *et al.*, 2010; Dunn *et al.*, 2011; Lai *et al.*, 2011; Cehn *et al.*, 2015). These organisms, in addition to *Kocuria*, are often considered contaminants especially when isolated from single positive blood culture sets since they are part of the normal microbiota of humans. The workup in many clinical microbiology laboratories is such that single positive blood cultures growing catalase-positive, coagulase-negative, Gram-positive cocci in clusters are identified minimally. Depending on laboratory protocols, a full species-level identification, which would be required to identify species of the genus *Kocuria*, only occurs when more than one blood culture set is positive or when requested by the ordering provider.

A total of 25 previous cases of human *Kocuria* infections have been reported in the literature. *K. kristinae* was first described in 1974 and has been reported to cause catheter-related bacteraemia (mostly central venous catheters) and infective endocarditis in immunocompromised hosts (Purty *et al.*, 2013). *K. kristinae* has also caused infection in a case of cholecystitis in an immunocompetent host as well as two cases of peritoneal dialysis-related peritonitis (Purty *et al.*, 2013; Cehn *et al.*, 2015; Ma *et al.*, 2005). In the paediatric population, *K. kristinae* has caused infections in premature babies and immunocompromised patients with long-term intravenous catheters for TPN and/or chemotherapy. A case of *K. varians* infection has been reported in a 52-year-old diabetic male who presented with a brain abscess (Tsai *et al.*, 2010). Other species of the genus *Kocuria* including *K. rhizophila*, *K.rosea* and *K. marina* have been described as aetiologic agents in various infections including a central venous line infection in a paediatric patient with methylmalonic aciduria, central line bacteraemia in a patient undergoing stem cell transplant, and cases associated with peritoneal dialysis (Savini *et al.*, 2010; Lee *et al.*, 2009; Altuntas *et al.*, 2005; Moissenet & Becker, 2012).

Due to the limited number of reports available, there are no guidelines for management of *Kocuria* infections or CLSI (Clinical & Laboratory Standards Institute) breakpoint interpretations for *in vitro* susceptibility testing of *Kocuria* isolates. Monotherapy with vancomycin, piperacillin/tazobactam, oxacillin or ciprofloxacin and combination therapy with teicoplanin and vancomycin, ciprofloxacin and clindamycin, and ceftriaxone and ofloxacin have been used successfully in case reports (Savini *et al.*, 2010; Purty *et al.*, 2013; Dunn *et al.*, 2011; Lai *et al.*, 2011; Cehn *et al.*, 2015). Previous reports have also suggested that removal of the intravascular catheters may be necessary for cure in cases of central venous catheter-associated bacteraemia, which suggests possible biofilm production by *Kocuria* (Dunn *et al.*, 2011; Lai *et al.*, 2011; Purty *et al.*, 2013; Cehn *et al.*, 2015). In our patients, vancomycin was used to treat the central venous catheter-associated bacteraemia infections. However, despite what was believed to be appropriate antimicrobial treatment (vancomycin MIC=0.5 mg ml^−1^ and 1.0 mcg ml^−1^), both patients continued to have positive blood cultures for *Kocuria* necessitating central line removal. Both patients cleared their bacteraemia after line removal and had favourable outcomes.

In conclusion, species of the genus *Kocuria* have been rarely reported to cause opportunistic infections in both adult and paediatric populations. It is likely that many laboratories are misidentifying species of the genus *Kocuria* as coagulase-negative *Staphylococcus* or *Micrococcus* due to the biochemical similarities among these species and laboratory protocols requiring minimal workup of these organisms. With newer organism identification methods, such as matrix assisted laser desorption/ionization time-of-flight (MALDI-TOF) mass spectrometry, becoming widespread across clinical microbiology laboratories, previously unknown pathogen-disease associations will be uncovered. The cases described here add to the growing body of literature documenting the pathogenicity of species of the genus *Kocuria* and the possible need for central line removal for achieving clinical cure in *Kocuria* central venous catheter-associated bacteraemia infections.
